# Functional balance associated factors in the elderly with chronic vestibular disorder

**DOI:** 10.1016/S1808-8694(15)31026-0

**Published:** 2015-10-19

**Authors:** Juliana Maria Gazzola, Monica Rodrigues Perracini, Maurício Malavasi Ganança, Fernando Freitas Ganança

**Affiliations:** a Physical therapist. Specialist in Gerontology - UNIFESP - EPM. MS in Sciences - Department of Otorhinolaryngology/Head and Neck Surgery - UNIFESP - EPM. FAPESP scholarship.; b Physical Therapist. PhD in Rehabilitation Sciences - UNIFESP - EPM. Professor, Coordinator of the Postgraduate couse in Physical therapy - City of São Paulo University - UNICID.; c Full Professor of Otorhinolaryngology - UNIFESP - EPM. Sernior Researcher - Stricto Sensu (MS) Postgraduate course in Neuro-Motor Rehabilitation Sciences - UNIBAN. Head of the Balance metrics Lab - Department of Otorhinolaryngology - Fleury Medical Diagnosis Center - São Paulo (SP).; d Otorhinolaryngologist - PhD in Medicine -UNIFESP - EPM (Associate Professor of Otoneurology - UNIFESP - EPM. Professor of the Postgraduate Stricto Sensu (MS) Program in Neuro Motor Rehabilitation UNIBAN. Head of the Vestibular Rehabilitation Sector - Discipline of Otoneurology - UNIFESP - EPM.)

**Keywords:** Elderly, Balance, Rehabilitation, Vestibular Disease, Dizziness

## Abstract

Daily activities can be challenging for the elderly.

**Aim:**

To study the association between functional balance, evaluated by the Berg Balance Scale (BBS), sociodemographics, clinical and mobilility (Timed up and go test - TUGT, Dynamic Gait Index - DGI) variables in the elderly with chronic vestibular disorder.

**Materials and Methods:**

A series study with one hundred and twenty elderly with chronic vestibular disorder. We performed the Mann-Whitney test, the Kruskal-Wallis test followed by Dunn test and the Spearman Coefficient (ρ).

**Results:**

Statistically significant associations and correlations were observed between total BBS score and age (ρ=-0.354; p<0.001), age group (p<0.001), number off illnesses (p=0.030), number of illnesses (ρ=-0.287; p=0.001), number of medications (p=0.014), number of medications (ρ=-0.274; p=0.002), recurrent falls (p=0.010), tendency to fall (p=0.002), topographic diagnosis of central vestibular disorder (p<0.001) and periodicity of dizziness (p=0.039), TUGT (ρ=-0.709; p<0.001) and DGI (ρ=-0.748; p<0.001).

**Conclusions:**

Functional balance in the elderly with chronic vestibular disorders evaluated by the BBS is worse when associated with aging, with a more advanced age group (80 years or more), increasing number of illnesses, presence of five or more illnesses, use of multiple medications, recurrent falls, tendency to fall, central vestibular syndromes, daily dizziness, mobility and gait impairments.

## INTRODUCTION

Population aging in Brazil is something that is happening in an accelerated fashion, causing a relevant increase in the prevalence of chronic-degenerative diseases^1^. This elderly population tends to have multiple diseases that have the potential do bring about major geriatric syndromes, such as falls, dementia, immobilization, which compromise both the independence and autonomy of these patients, causing disabilities, frailty, institutionalization and death^1^, ^2^, ^3^.

Vestibular dysfunction takes on a particularly relevant role, since age increase is directly related to the presence of multiple associated otoneurological symptoms such as vertigo and other types of dizziness, hearing loss, tinnitus, body balance alterations, gait disorders and occasional falls, among others^4^. Tinetti et al.^5^ have considered diziness as a geriatric syndrome, of multifactorial cause that happens because of the build up effect of deficits from multiple systems, causing greater vulnerability in the elderly to daily challenges. Posture control may be influenced by the very physiologic alterations pertaining to aging, chronic diseases, pharmacologic interactions or specific dysfunctions. The aging process affects all postural control components - sensorial (visual, somatosensorial and vestibular), effector (strength, range of motion, biomechanical alignment, flexibility) and central processing^6^. The integration of the different body systems under central command is critical for body balance control. The performance of such systems has a direct impact on the individual's capacity to perform daily tasks, his/her functional capacity^7^.

One of the simpler ways to check the involvement of these systems, of which integrity is fundamental for the normal performance of motor tasks, is the functional assessment, which simulates exactly those demands involved in the capacity to control balance, and it may be useful in order to consider the determining factors of the observed functional limitation determined^8^. The Berg Balance Scale (BBS) is a balance functional assessment tool that is very much used for both clinical and research purposes validated by Berg et al.^9^ and culturally adapted to be used in Brazil^10^, ^11^. The test is made up of 14 tasks, each classified in a five point ordinal scale, which ranges from zero (unable to perform the task) to five (performs the task all by him/herself), based on performance quality, need for help and the time needed to complete the task. Scores of the 14 tasks are added up into a total score that varied between 0 and 56, and the highest score is related to a better performance. It is easy to be managed and safe for the elderly patients submitted to the assessment. It serves numerous needs, such as balance skill qualitative description; follow up of the patients’ clinical development, and therapeutic intervention effectiveness^12^. Medeiros^13^ found the cutting point score to be of 48 on the BBS for fall forecasting, when he assessed 76 elderly patients with vestibular disease complaining of chronic diziness. He achieved a specificity of 58.0%, sensitivity of 78.0%, Predictive Positive Value of 72.9%, Predictive Negative Value of 57.5% and 69.7% accuracy. Cutting point 48 on the BBS had greater clinical relevance and is more precise in relation to other cutting points in finding those that fall within this elderly population with vestibular diseases.

The lack of knowledge about balance functional characteristics in the elderly with chronic vestibular dysfunction and the identification of balance deficit associated variables in these individuals may cause the development of specific prevention strategies, rehabilitation support, aiming at maintaining the autonomy and, thus, preserve the person's independence for the longest possible time. We did not find in the literature we consulted, any study on the balance functional assessment in the elderly with chronic vestibular dysfunction who have been assessed by means of the BBS, in order to identify the factors associated to functional balance in this population. Although it might be a consensus that sensorial integration assessment and dynamic control by means of the posturography protocols be more detailed for the detection of balance components which are more affected, understanding the functional performance of posture adjustments may show, in a simple and low cost way, changes that imply the practice of a therapeutic intervention used in order to improve functional capacity^8^. The goal of the present study is to check the association between the functional balance, assessed by the Berg Balance Scale (BBS), and the socio-demographic, clinical and mobility data (Timed Up and Go Test - TUGT) and gait (Dynamic Gait Index - DGI) of elderly patients with chronic vestibular dysfunction.

## METHOD

This is an analytic transversal study, previously approved by the Research Ethics Committee of the Federal University of São Paulo - Paulista School of Medicine (UNIFESP - EPM), protocol # 0371/03. All the patients included in this study read the Information Letter and signed the Informed Consent. This is part of a study that was financed by the Fundação de Amparo à Pesquisa do Estado de São Paulo (FAPESP), protocol # 03/10119-3.

The sample was comprised of patients from the Otoneurology Department UNIFESP - EPM, aged 65 or more, males or females, with chronic vestibular dysfunction, characterized by a complaint of dizziness and/or balance loss, stunning and/or other inespecific dizziness sensations, for at least three months. We exclude those elderly patients who had physical, cognitive and sensorial limitations which prevented them from undergoing the balance tests, such as incapacity to understand and obey a simple verbal command and/or mimic movements; those with severely impaired visual and hearing acuities and absolutely disabling to daily activities, even when wearing corrective lenses and/or hearing aids; those with lower and upper limb amputations; those unable to walk independently and those who were able to move only on a wheelchair. We also excluded those patients who had undergone some kind of body balance rehabilitation program in the 6 months prior to the assessment. The assessment was not carried out during the dizziness spell.

The elderly were initially submitted to a clinical otoneurological assessment, which included otorhinolaryngological interview and physical exam, audiometry, immitanciometry and vestibular tests, carried out by means of the vectoelectronystagmography, according to the criteria proposed by Ganança et al.^14^ and Ganança et al.^15^. The data was collected in medical offices, broad halls and stairways located in our department between April of 2003 and November of 2004. The socio-demographic data attained were: gender, age (complete years of life) and age range. The clinical data analyzed were number of diseases, number of medications, possibility of falls and recurrent falls in the previous year, syndromical and topographic diagnosis and topodiagnosis of the vestibular dysfunction, number of vestibular affections associated, dizziness time of onset, dizziness type, dizziness duration and dizziness frequency. Balance clinical evaluation was determined by the use of balance functional scales: BBS^11^, ^12^ and Timed Up and Go Test^16^. In order to assess balance during gait we also used the Dynamic Gait Index^17^ tool.

For BBS^11^, ^12^ we used the cutting value of 48, as a predictor for vestibular diseased patients’ falls, according to Medeiros^13^. A score of 19 or less in DGI is related to falls in the elderly^18^. We found a significant correlation between the score achieved in this tool and the BBS score (r=0.71; p<0.01), in other words, the higher the DGI score, the higher the BBS score^19^. TUGT has a strong association with the BBS score and is sensitive to changes along time. Podsiadlo and Richardson^16^ considered a time of up to 10 seconds as a good performance for normal and healthy adults; between 11 and 20 seconds is expected for frail elderly or those with disabilities, who tend to be independent for most daily life activities; above 20 seconds spent to perform a task suggests an important loss in mobility, making it necessary to have a more detailed assessment. The material used were one chair with arm support, one chair without arm support, a 20.5cm step, measuring tape, chronometer (Casio®), an obstacle (caixa de sapato) and two traffic signaling cones.

For inferring statistical analysis of the present study the total BBS score was compared among the variables by means of the Mann-Whitney and Kruskal-Wallis, followed by the Dunn test and by the Spearman correlation coefficient (ρ). We used he non-parametric tests due to the asymmetry and variability of the BBS score and the lack of normal distribution (p < 0.01). The significance level used for the statistical tests was of 5% (α=0.05). The analysis was carried out by the SAS System for Windows (Statistical Analysis System, software, version 6.12^20^.

## RESULTS

Our sample was made up of 1020 elderly patients with a medical diagnosis of chronic vestibular syndrome, being followed up in our ward, 68.3% females and 31.7% males, with average age of 73.40 years and standard deviation (SD)^5^^,77^. Complete otoneurological, clinical-functional and socio-demographic information of this sample is described in another publication^21^.

**Figure 1:**
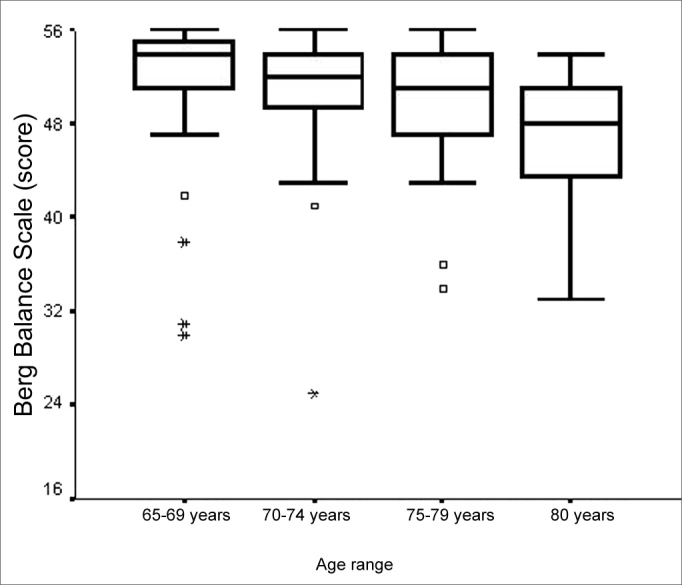
Graphic representation of the Berg Balance Scale variable in function of the variable category “age range” of elderly people with chronic vestibular disorder (n=120).

**Figure 2:**
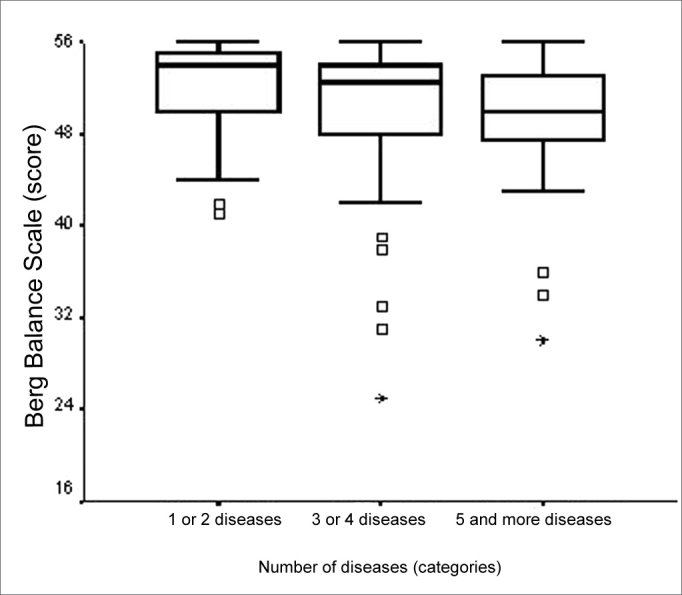
Graphic representation of the Berg Balance Scale variable in function of the variable category “number of diseases” in elderly people with chronic vestibular disorder (n=120).

We noticed a week negative statistically significant correlation between BBS score and patient age (ρ= -0.354; p<0.001). We also noticed an association between BBS score and age range (p<0.001). The major differences were seen among the age range medians”65 to 69 years” and “80 years or more” and “70 to 74 years” and “80 years or more” (Figure 1), and the values of the total BBS score medians found were lower for the highest age range - 80 years and above. We did not find significant associations between total BBS score and gender. There was a negative and week statistically significant correlation between total BBS score and number of diseases (ρ= -0.287; p= 0.001) and also a significant association between total BBS score and the category variable “number of diseases” (p=0.030). The main difference happened among the total BBS score medians in the categories of “1 or 2 diseases” and “5 or more diseases” (Figure 2), being lower in the elderly with more diseases associated.

**Figure 3:**
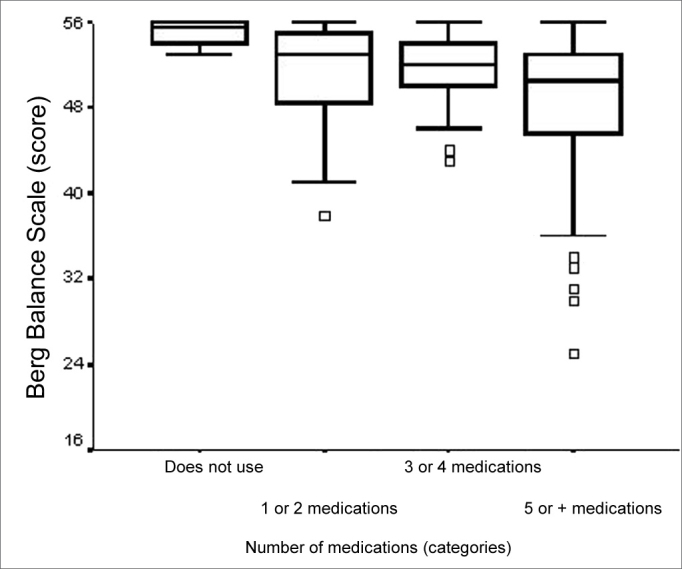
Graphic representation of the Berg Balance Scale variable in function of the variable categories.

Total BBS score presented a weak statistically significant correlation with the number of medications used (ρ= -0.274; p= 0.002). There also was an association between total BBS score and the number of medications (p=0.014). The main differences were seen between the medians of the “does not use” and “3 or 4 medications” and “does not use” and “5 or more medications” categories (Figure 3). We noticed that the elderly that used more medication had a worse balance performance when compared to their counterparts who did not use medication.

**Figure 4:**
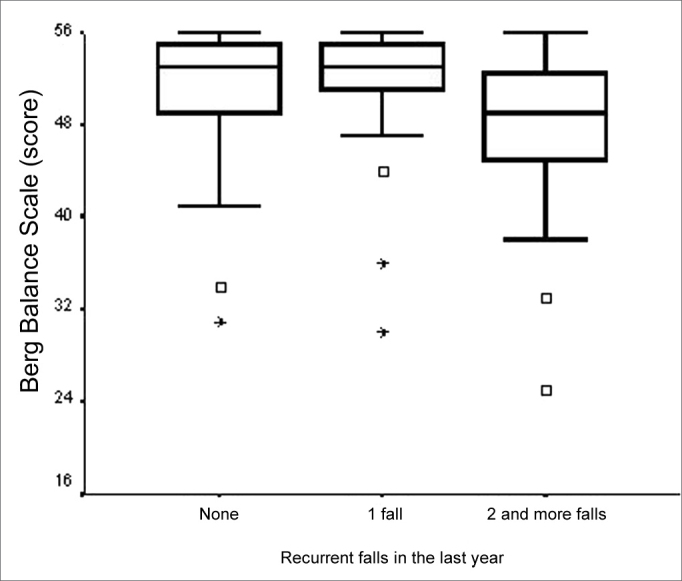
Graphic representation of the Berg Balance Scale variable in function of the variable categories

Concerning falls that had been reported for the previous year, we did not find statistically significant differences between vestibular impaired elderly BBS score who had falls, when compared to those who did not fall. BBS score was related to the “number of falls” of the variable bearing three categories (none/1 fall/two or more falls), with descriptive level significantly equal to 0.010. The greatest differences were found among medians of the “no fall” and “two or more falls” and “one fall” and “two or more falls” categories (Figure 4). BBS median values were equal in the categories “no falls” and “one fall”, and lower in the category “two or more falls”.

**Figure 5:**
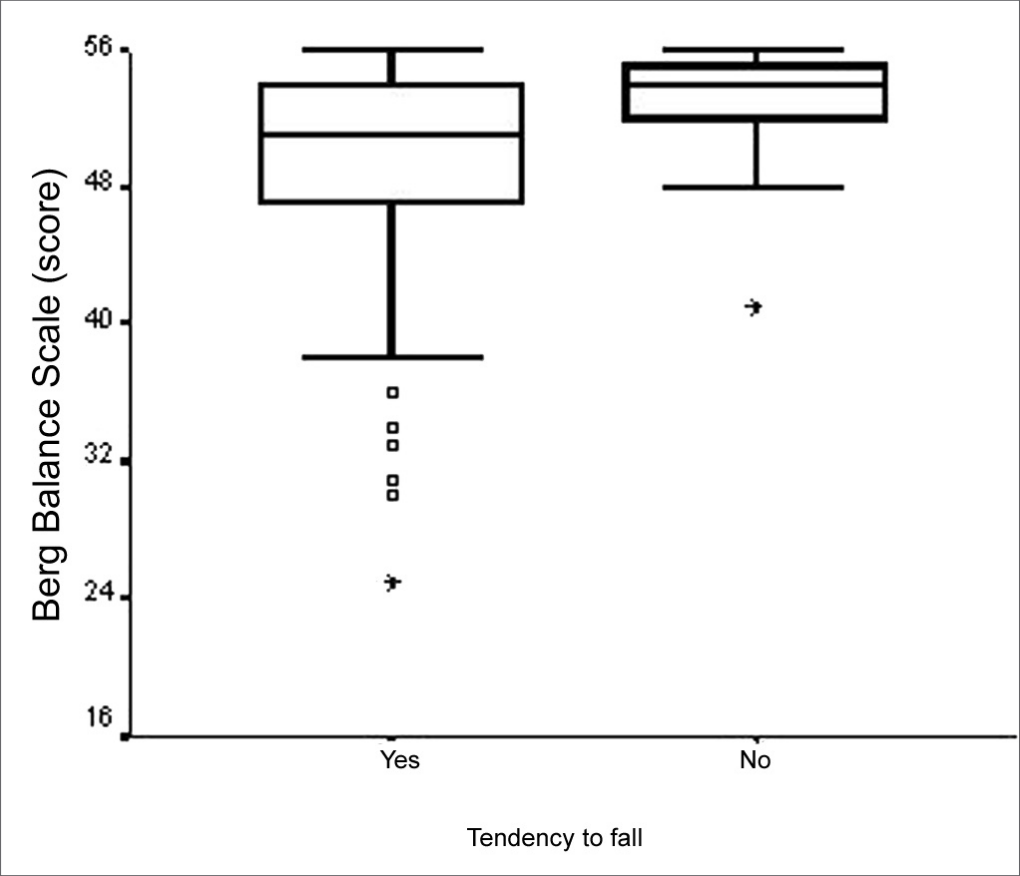
Graphic representation of the Berg Balance Scale variable in function of the variable category “tendency to fall” in elderly people with chronic vestibular disorder (n=120).

**Figure 6:**
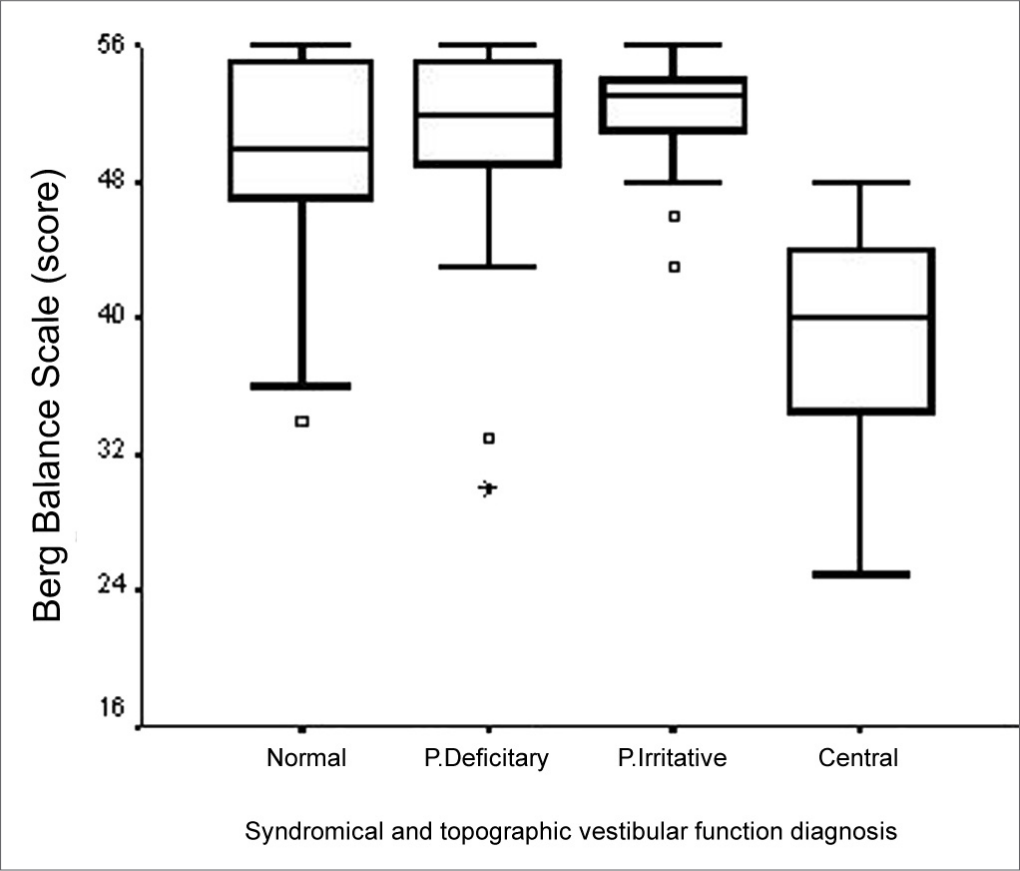
Graphic representation of the Berg Balance Scale variable in function of the variable category “vestibular dysfunction topographic and syndromical diagnosis” in elderly people with chronic vestibular disorder (n=120).

Total BBS score was significantly lower for those elderly with chronic vestibular syndromes who reported a fall tendency (p=0.002), when compared to those who did not report it (Figure 5). In analyzing the category variable “Vestibular Dysfunction topographic and syndromical diagnosis” in relation to total BBS score, there was a statistically significant difference (p<0.001). The major differences happen among the “normal” and “central”; “deficitary peripheral” and “central”; and also between “irritative peripheral” and “central” categories (Figure 6). The elderly with a central vestibular syndrome topographic diagnosis, given by the vestibular test, had a worse performance in the BBS functional balance assessment. We noticed that the number of concurrent vestibular disorders was not related to the total BBS score.

**Figure 7:**
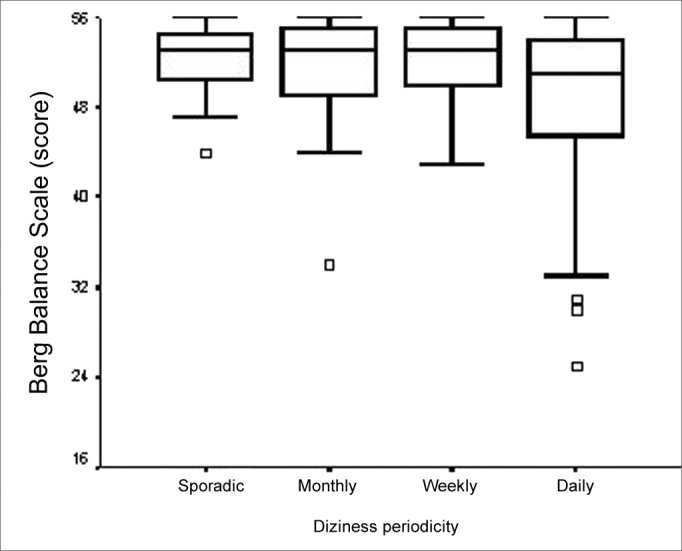
Graphic representation of the Berg Balance Scale variable in function of the variable category “vestibular dysfunction topographic and syndromical diagnosis” in elderly people with chronic vestibular disorder (n=120).

Total BBS score was not related to the “time of dizziness onset”, “type of dizziness” and “dizziness duration” variables. Notwithstanding, BBS did point an association with “dizziness frequency” (p=0.039), in which the main difference happened between the “daily” and “sporadic” categories. The elderly who reported daily dizziness had a lower value on the BBS median (Figure 7).

Representation of the Berg Balance Scale variable in function of the variable category “diziness frequency” in elderly people with chronic vestibular disorder (n=120).

**Figure 8:**
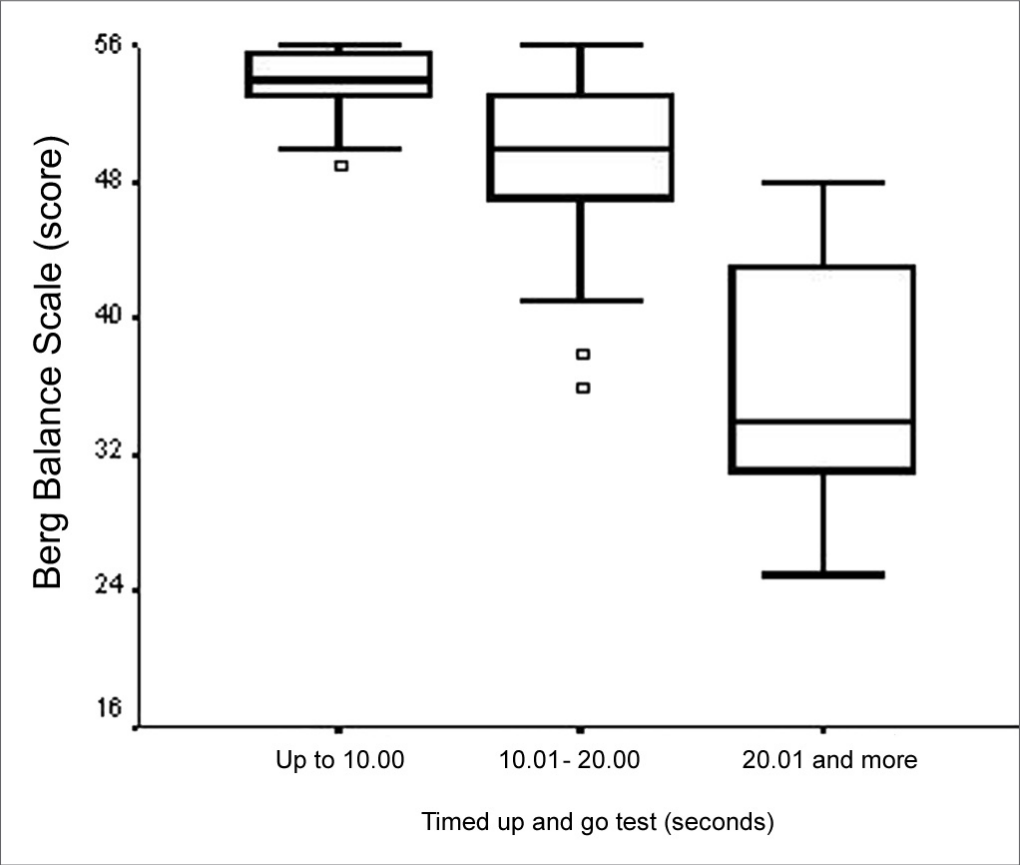
Graphic representation of the Berg Balance Scale variable in function of the variable category “Timed Up and Go Test” in elderly people with chronic vestibular disorder (n=120).

**Figure 9:**
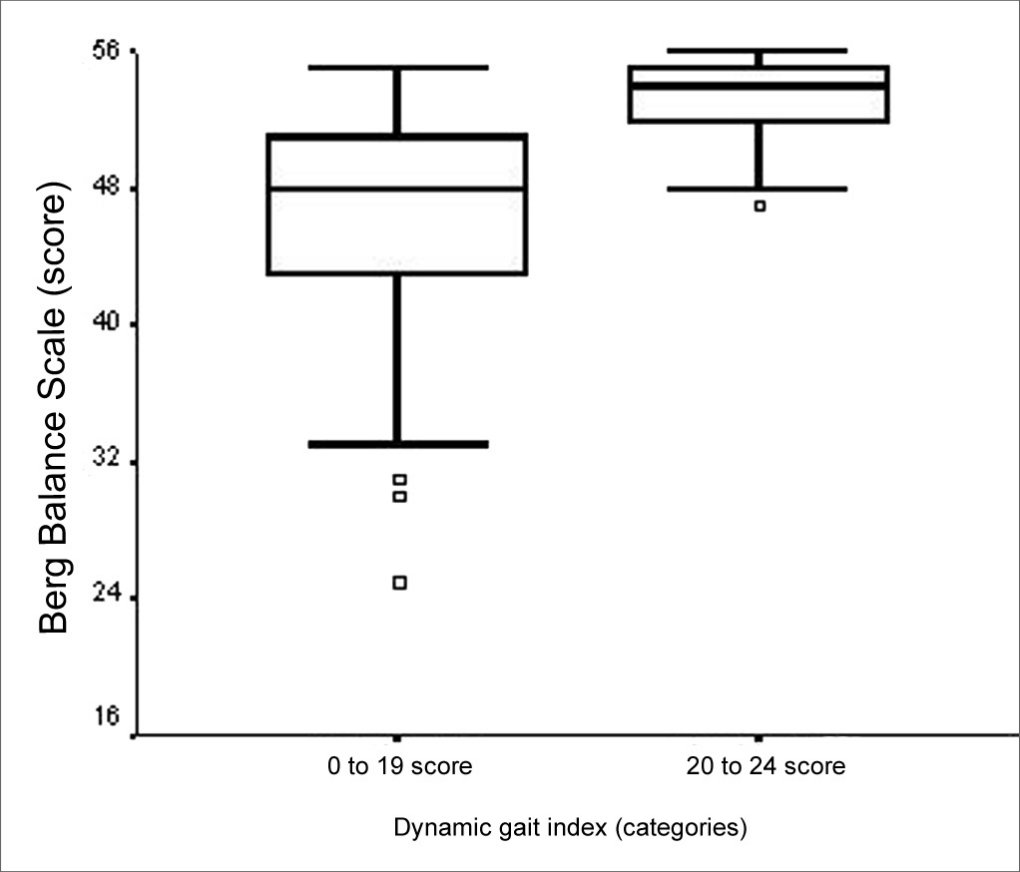
Graphic representation of the Berg Balance Scale variable in function of the variable category “Dynamic Gait Index” in elderly people with chronic vestibular disorder (n=120).

Both TUGT and BBS total scores presented a moderate negative statistically significant correlation (ρ=- 0.709; p<0.001). We also found an association when the analysis was carried out considering total BBS score and the three time categories defined (p<0.001). We found the differences among the medians of the three TUGT execution time span categories and the BBS score attained among the “up to 10.0 seconds” and “10.01 to 20.0 seconds” and among the “20.01 seconds and more” category and the other two time categories (Figure 8). We observed that the higher the BBS score, lower is the time spent in the TUGT performance. In assessing balance during gait, through DGI, the total score of this tool presented a moderately statistically significance with total BBS score (ρ= -0.748; p=0.001). Total BBS score also presented a significant difference among the medians of the “0 to 19 points” and “20 to 24 points” categories (Figure 9), BBS score was lower in the “0 to 19” category (p<0.001).

## DISCUSSION

The development of a study geared towards providing some understanding of the functional balance in the elderly with chronic vestibular disorder can make the specialist more attentive to a greater number of associations between functional limitations and disabilities or incapacities, allowing for a more precise functional diagnosis in rehabilitation and a better monitoring of the clinical picture development.

In the present study we observed that the BBS score median was significantly lower for the elderly at a higher age range, in other words, those with 80 years of age or more, probably because the involvement of the systems responsible for body balance is proportional to an increase in age^22^, ^23^. We also found a significant correlation between the patient's age and total BBS score, and the latter was reduced with an increase in age.

Miyamoto^10^ also found a significant correlation (r= -0.353 p<0.05) in a sample of 36 patients with 65 years of age or more, who presented higher prevalence of rheumatologic, cardiovascular and bone disease (osteoporosis). We did not find statistically significant difference between the BBS score and gender, in agreement with Miyamoto^10^.

We noticed a worse functional balance performance in the elderly affected by five or more diseases and also that the more diseases the patient had the less functional balance performance was seen in the elderly who had alterations in their vestibular system. BBS score was not related to the number of diseases in the study by Gazzola et al.^24^, who studied elderly patients in a gerontology rehabilitation service, who had a greater prevalence of cardiovascular and bone/joint diseases, in whom the otoneurological disorders corresponded to only 21.5%. Such finding allows us to infer that the presence of vestibular disorders in elderly patients impairs balance and increase the risk of falls^25^, specially in those elderly patients with more diseases. As it was expected, elderly patients with central vestibular disorders in the present study showed a worse balance performance on BBS, because of the greater possibility of involvement of other areas of the central nervous system, related to body balance, the posterior fossa (brain stem and cerebellum) for example or at a cortical level^26^.

We found a statistically significant correlation between the lower total BBS score and the wider use of medication. There was a statistically significant association between total BBS score and the groups of elderly patients who took “3 or 4 medications” and “5 or more medications”, when compared to the ones who did not do it. These results are in agreement with the ones from Sloane et al.^27^ and Ganança et al.^28^ who stressed that poly-pharmacotherapy may increase the risk of drug interaction and that of adverse effects, and it usually worsens labyrinth symptoms and, just like Tinetti et al.^5^ who showed that the use of five or more drugs together is associated to a greater risk of dizziness in the elderly.

A significant association was found in the current study between BBS score and recurrent falls, in which the elderly with past history of two or more falls presented more functional balance impairment. In the study carried out by Berg et al.^29^, BBS proved to e relevant as a predictor factor for multiple falls, in up to 2.7 times the cutting point^45^. Notwithstanding, Miyamoto^10^ did not find significant correlations between BBS score and the number of falls the elderly had. In the present study we observed that the BBS measure medians of the elderly who fell was equal to that of the elderly who did not fall. This allowed us to state that BBS is unable to differentiate patients who fell from those who did not and that such classification only differentiated patients as to their increased functional disability, such as, for example, those with past history of recurrent falls. In the present study, fear of falling and a tendency to fall were reported by most of the patients, 72.5% and 79.2%, respectively. We also found an association between the worst functional balance performance and a tendency to fall. Such findings corroborate the studies by Tinetti et al.^30^ and Lachman et al.^31^, who observed that the fear of falling causes consequent mobility impairment, thus compromising the body balance performance of the elderly with vestibular disease, with a greater tendency to fall.

The elderly who reported daily dizziness had worse balance performance when compared to those who reported sporadic dizziness spells, probably due to a greater limitation caused by dizziness, which is more frequent.

As expected, we noticed a moderate correlation between TUGT performance worsening and a BBS performance worsening, already reported by Podsiadlo and Richardson^16^ and Berg et al.^9^ in elderly people of the community, by Cordeiro ^8^ in elderly with diabetes mellitus under outpatient follow up and by Gazzola et al.^24^ in patients from gerontology rehabilitation. It is important to highlight that the BBS does not assess aspects deemed important for balance as in gait dynamic balance, attention aspects, postural responses in unstable surfaces and postural responses to external changes such as the reactive strategic changes in ankles, hips, trunk and the back step. It is known that the assessment of those elderly patients who are have some balance-related complaint should be carried out in the most complete possible way, using various tools^8^, ^10^, ^12^, ^24^, ^32^.

One of the limitations of the present study was that we did not assess some sensorial components of the postural control such as visual acuity, postural-kinetic propioceptive sensitivity skin protection sensitivity, vibration sensitivity, sensorial interaction tests and effector components such as muscle power, connective tissue and muscles flexibility and range of motion. Research involving such information about vestibular impaired elderly patients should also be carried out in order to broaden current knowledge about body balance in these patients. And finally, we stress the importance of carrying out studies with longitudinal follow up of elderly patients with chronic vestibular dysfunction submitted to many therapeutic options, in order to establish more efficient treatment means, avoid scaling up functional limitations ameliorate or delay progressive degeneration processes and improve the life quality of such patients.

## CONCLUSION

Functional balance of elderly patients with chronic vestibular dysfunction, assessed by BBS is worse when associated to advancing age, the older age range (80 years or more), increase in the number of diseases, presence of five or more diseases associated to the vestibular disorder, poly-pharmacotherapy, recurrent falls, tendency of falls, central vestibular syndromes, daily diziness, compromised mobility and gait disorder.
